# Berberine produces antidepressant-like effects in ovariectomized mice

**DOI:** 10.1038/s41598-017-01035-5

**Published:** 2017-05-02

**Authors:** Jie Fan, Bingjin Li, Tongtong Ge, Zhuo Zhang, Jiayin Lv, Jing Zhao, Pu Wang, Wei Liu, Xuefeng Wang, Katarzyna Mlyniec, Ranji Cui

**Affiliations:** 1 Jilin Provincial Key Laboratory on Molecular and Chemical Genetics, The Second Hospital of Jilin University, Changchun, Jilin 130041 China; 2 Department of Orthopedics, China-Japan Union Hospital of Jilin University 126 Xiantai Street, Nanguan District, Changchun 13033 China; 3 School of Life Sciences, Northeast Normal University, Changchun, Jilin 130024 China; 4 Department of Pharmacobiology, Jagiellonian University Medical College, Medyczna 9, PL 30-688 Krakow, Poland

## Abstract

Berberine has been reports to have antidepressant-like effects. However, it is seldom known whether berberine produces antidepressant-like effects in ovariectomized mice, which exhibit depressive-like responses. To examine the antidepressant-like effects of berberine in ovariectomized mice, behavioral tests were conducted, including the forced swimming test and the open field test. To elucidate the mechanisms, levels of BDNF, phosphorylated CREB and phosphorylated eEF2 were analyzed by western blotting, and c-Fos induction was examined by immunohistochemistry. In the forced swimming test, berberine decreased the immobility time in a dose-dependent manner, reversing the depressive-like effect observed in ovariectomized mice, and this effect was blocked by the 5-HT_2_ antagonist ketanserin. In addition, western blotting indicated that BDNF and peEF_2_ in the hippocampus, but not pCREB/CREB in the frontal cortex, were affected by berberine treatment. Furthermore, immunohistochemistry demonstrated that the reduction in c-Fos induced by ovariectomy were greater after berberine treatment. Ketanserin also antagonized the effect of berberine on the c-Fos expression. Our findings suggest that berberine exerts antidepressant-like effects in ovariectomized mice, and 5-HT_2_ receptor activation may be partially related to the antidepressant-like effects of the berberine by BDNF-CREB and eEF_2_ pathways.

## Introduction

Depression is the most common mental disorders in humans. Although two-thirds of patients recover from a depressive episode spontaneously, the remaining one-third cannot recover without antidepressant treatment^1–3^. Many antidepressant treatments are currently available, but they are not effective in all individuals, Furthermore, they are associated with adverse effects in many individuals. Therefore, the present study aimed to identify a new potential antidepressant drug with fewer side effects. In our previous study the total Fuzi alkaloid extract produces antidepressant-like effects^4^. Therefore, in this study, the antidepressant-like effect of berberine, another specific alkaloid, was investigated in ovariectomized (OVX) mice.

Berberine is an isoquinoline quaternary alkaloid that can be exacted from many medicinal plants^5, 6^. It has several known pharmacological effects^7–16^. In particular, berberine inhibits corticosterone-induced depressive-like behavior in mice, which may be due to up-regulates BDNF expression^13^. Another report demonstrated that berberine could exert antidepressant-like action in both the forced swimming test and the tail suspension test with elevated 5-HT/NE/DA levels^14^. However, the antidepressant-like effect of berberine in OVX mice has rarely been studied.

Eukaryotic elongation factor 2 (eEF_2_) is involved in the actions of rapid-onset antidepressants^17, 18^. It has been reported that rapid-onset antidepressant-like behavior is mediated by the decreased phosphorylation of eEF_2_ and the increased translation of BDNF^17, 18^. Other studies have demonstrated that the CREB and phosphorylated CREB (pCREB) levels are upregulated in rodent brains during antidepressant treatment^19–21^. The BDNF-CREB pathway is a well-established antidepressant pathway that plays a critical role in antidepressant action^20, 22, 23^. Furthermore, it is has been reported that antidepressant treatment increase c-Fos expression^24–27^. Therefore, the present study was aimed to examine the antidepressant-like effect of berberine in OVX mice. The effect of berberine on the BDNF-CREB-eEF2 pathway was analyzed, including signaling mediated by pCREB and phosphorylated eEF2 (peEF2), which was assessed using the pCREB/CREB and peEF2/eEF2 ratios. Immunohistochemical staining of c-Fos was used to test the antidepressant-like action of the berberine.

## Results

### Effect of repeated berberine treatment on body weight

**Figure 1:**
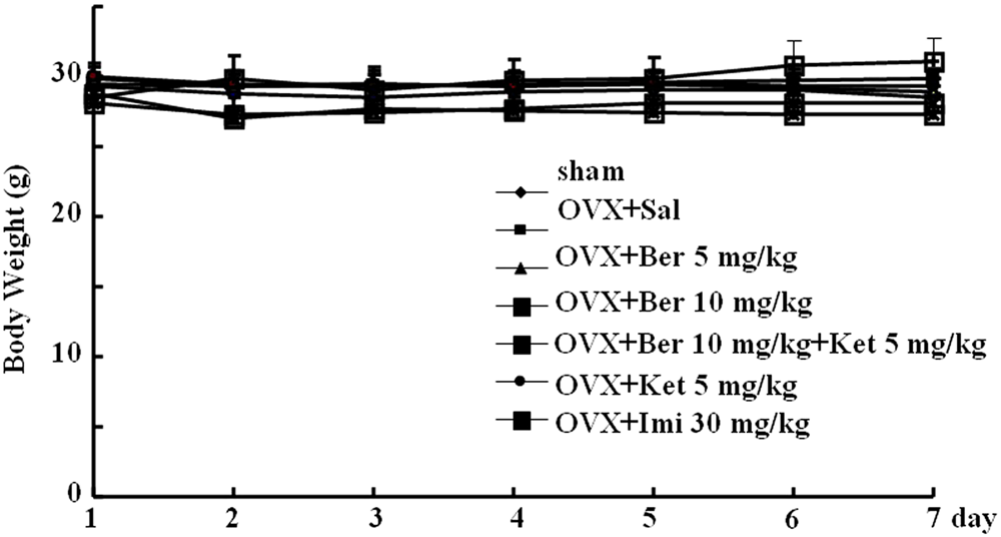
Body weight in subjects from all groups over the 7-day course of pharmacological treatment. sham-treated mice (sham) and ovariectomized (OVX) mice treated with saline (OVX + Sal), 5 mg/kg berberine (OVX + Ber 5 mg/kg), 10 mg/kg berberine (OVX + Ber 10 mg/kg), 10 mg/kg berberine + ketanserin (OVX + 10 mg/kg Ber + Ket, ketanserin only (OVX + Ket 5 mg/kg), or imipramine (OVX + Imi 30 mg/kg).

Repeated administration of berberine or imipramine did not change the body weight of OVX mice, nor did any of the other treatments. A two-way ANOVA revealed no significant effect of drug treatment (*P* > 0.05), and no significant interaction between the treatment and time (*P* > 0.05) (Fig. 1).

### Effect of repeated berberine treatment on locomotor activity

**Figure 2:**
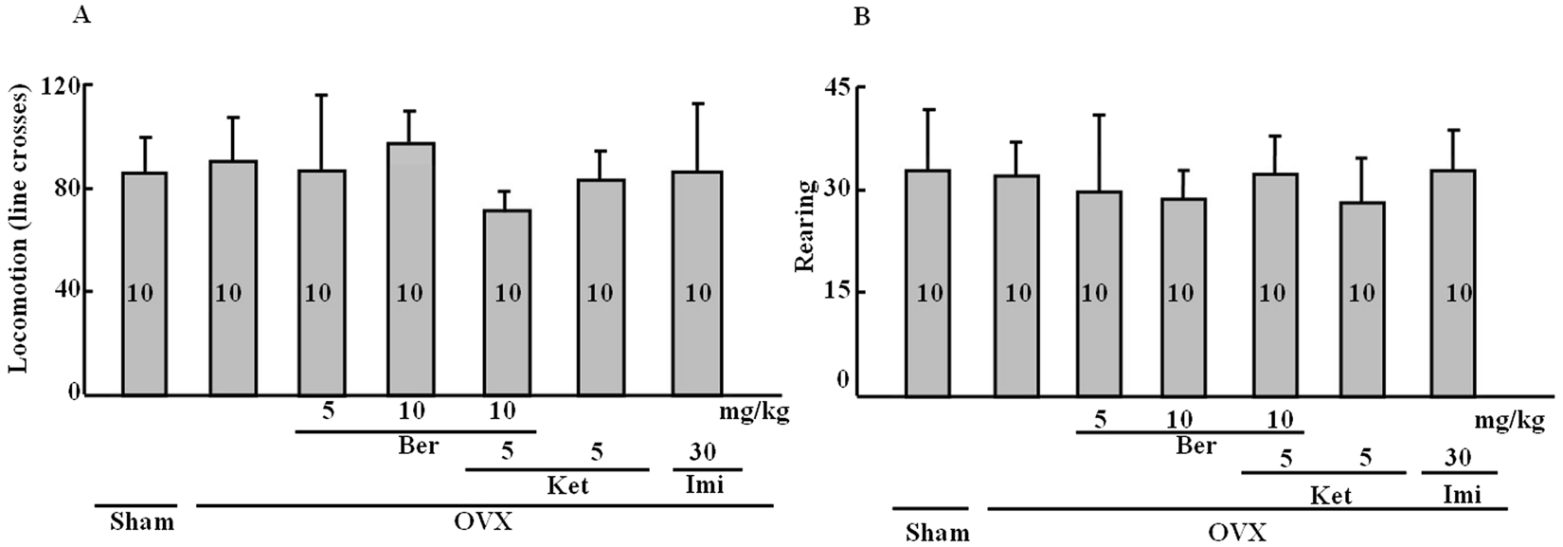
Locomotor behavior and rearing behavior in the open field. (**A**) Locomotor behavior (number of line crosses) in the open field. (**B**) Rearing behavior (frequency) in the open field. Group conditions are indicated by abbreviations, and doses by numbers: Sham (sham treatment), OVX (ovariectomy), Ber (berberine, 5 or 10 mg/kg), Ket (ketanserin, 5 mg/kg) and Imi (imipramine, 30 mg/kg).

Figure 2a and b reveal that berberine did not affect locomotor activity or rearing (locomotor activity: *P* > 0.05; rearing: *P* > 0.05). This indicates that general changes in activity were not responsible for the differences in immobility time induced by berberine in the forced swim test.

### Effect of repeated berberine treatment on immobility time

**Figure 3:**
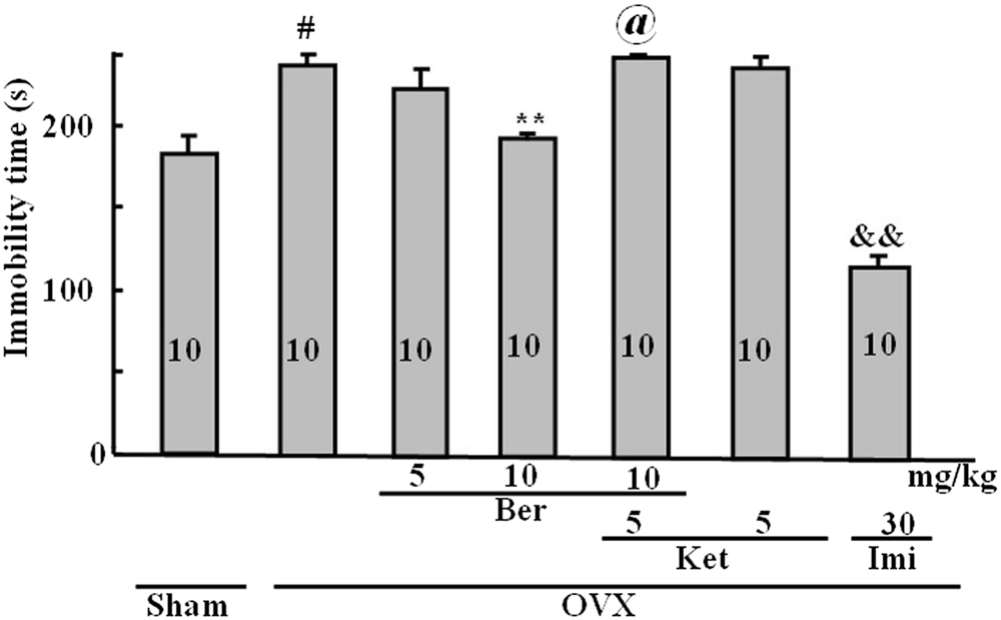
Duration of immobility time (s) in the forced swim test. Group conditions are indicated by abbreviations, and doses by numbers: Sham (sham treatment), OVX (ovariectomy), Ber (berberine, 5 or 10 mg/kg), Ket (ketanserin, 5 mg/kg) and Imi (imipramine, 30 mg/kg). Symbols represent significant *post hoc* comparisons: Tukey’s HSD, ^#^ *P* < 0.05 vs. sham, **P < 0.05 vs. OVX, ^@^ *P* < 0.05 vs. OVX + Ber 10 mg/kg, ^&&^ *P* < 0.01 vs. OVX.

Figure 3 shows that immobility time was significantly change across treatment groups (treatment effect: *P* < 0.001). Immobility time was increased in OVX mice compared to the sham treatment group (*P* < 0.01), and berberine dose-dependently decreased the ovariectomy-induced increase in immobility time. Berberine (10.0 mg/kg) produced a marked reduction compared to the OVX only group (*P* < 0.01). Interestingly, ketanserin (5.0 mg/kg) reversed the effect of berberine on immobility time (*P* < 0.001). This reversal normalized immobility compared with the sham control group. Imipramine significantly inhibited the immobility time compared with the OVX only group (*P* < 0.01). However, ketanserin alone had no effect.

### Effect of the repeated berberine treatment on the pCREB/CREB ratio

**Figure 4:**
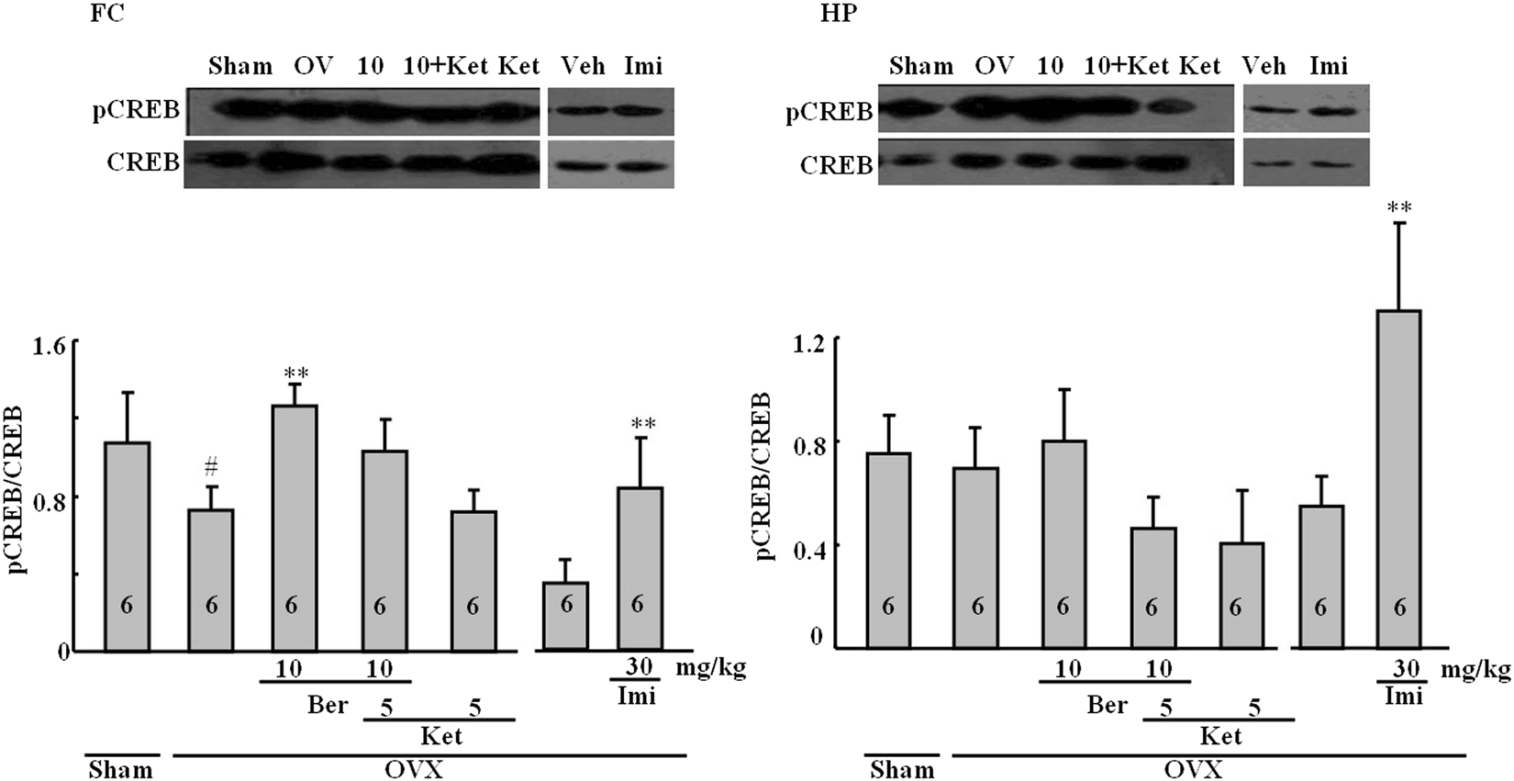
Representative western blots for CREB and pCREB. The frontal cortex (FC; left) and the hippocampus (HP; right). Figures represent the pCREB/CREB ratios in the FC and HP. Group conditions are indicated by abbreviations and doses by numbers: Sham (sham treatment), OVX (ovariectomy), Ber (berberine, 5 or 10 mg/kg), Ket (ketanserin, 5 mg/kg) and Imi (imipramine, 30 mg/kg). Symbols represent significant *post hoc* comparisons: Tukey’s HSD, ^#^ *P* < 0.05 vs. sham, **P < 0.05 vs. OVX only.

The western blotting results for pCREB and CREB, with averages for each treatment group, are shown in Fig. 4. Differences in the pCREB/CREB ratios were observed in the frontal cortex (*P* < 0.05), but not in the hippocampus (*P* > 0.05). Ovariectomy reduced the pCREB/CREB ratio (*P* < 0.05). This effect was reversed by berberine (*P* < 0.05), whereas the berberine/ketanserin group was not much different from the OVX-only group. Imipramine produced an elevation in the pCREB/CREB ratios in the hippocampus and the frontal cortex (*P* < 0.05).

### Effect of the repeated berberine treatment on the BDNF protein level

**Figure 5:**
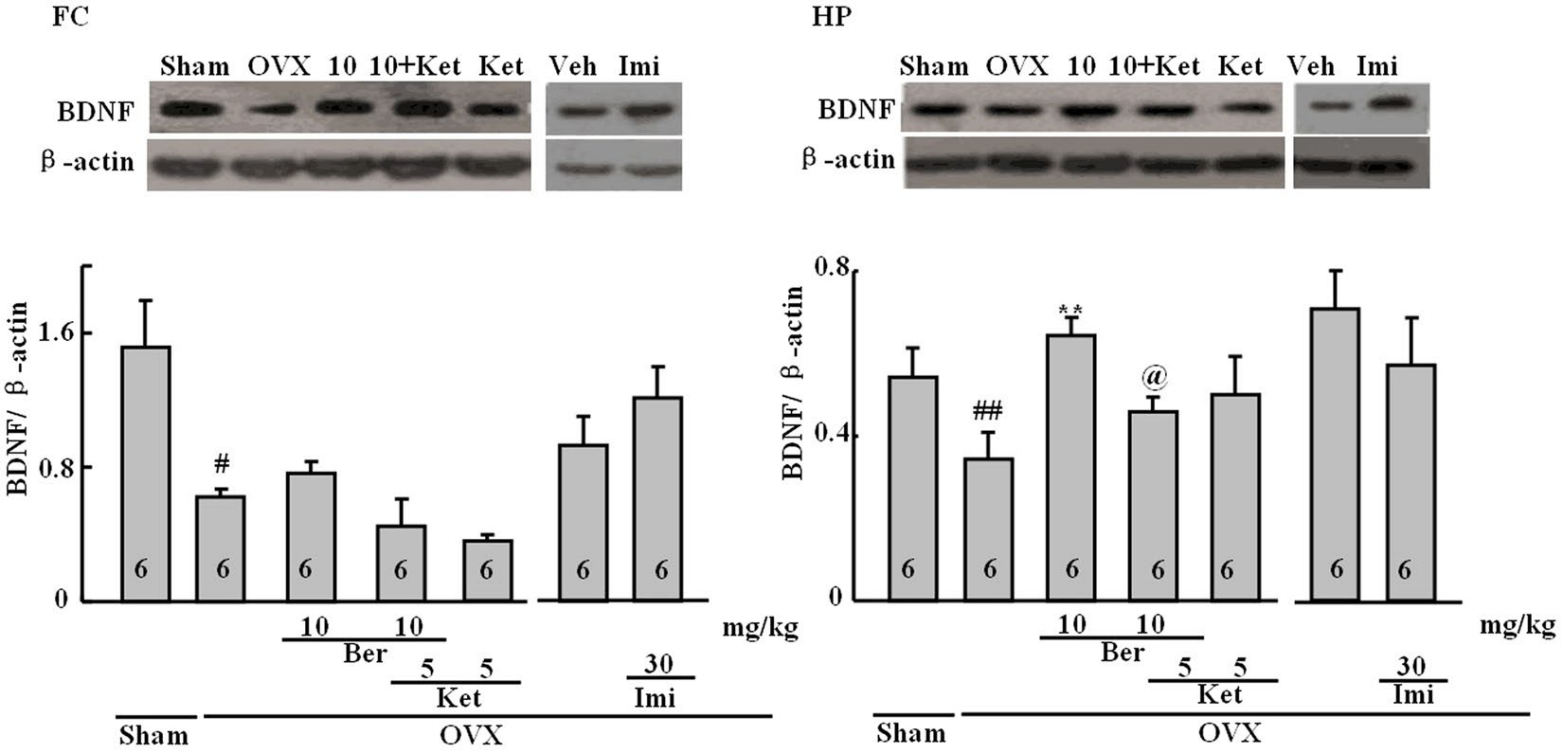
Representative western blots for BDNF and β-actin. The frontal cortex (FC; left) and the hippocampus (HP; right). Figures represent the BDNF levels (normalized to β-actin) in the FC and HP. Group conditions are indicated by abbreviations and doses by numbers: sham (sham treatment), OVX (ovariectomy), Ber (berberine, 5 or 10 mg/kg), Ket (ketanserin, 5 mg/kg) and Imi (imipramine, 30 mg/kg). Symbols represent significant *post hoc* comparisons: Tukey’s HSD, ^#^ *P* < 0.05 vs. sham, **P < 0.05 versus OVX only.

Substantial differences in the levels of BDNF (normalized to β-actin levels) were observed across treatment groups in both the frontal cortex (*P* < 0.001) and the hippocampus (*P* < 0.05), as shown in Fig. 5. Ovariectomy reduced the BDNF levels in both the hippocampus (*P* < 0.01) and the frontal cortex (*P* < 0.001). Berberine normalized the BDNF levels in the hippocampus (*P* < 0.01 vs. OVX alone, Tukey’s HSD) and this effect was reversed by ketanserin, which was not different from the OVX group. Imipramine did not affect the BDNF levels in either the hippocampus or frontal cortex

### Effect of the repeated berberine treatment on the peEF_2_/eEF_2_ ratio

**Figure 6:**
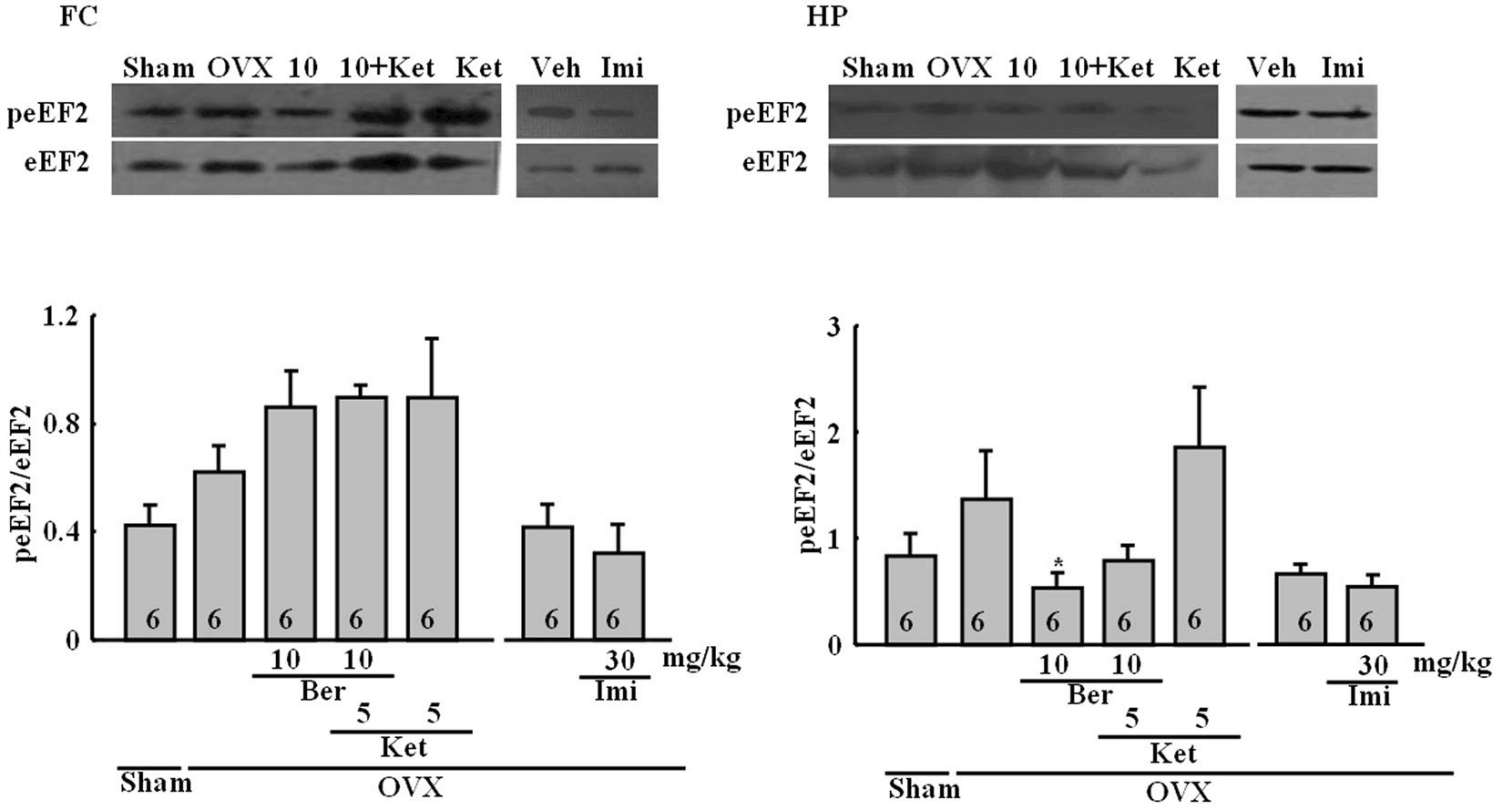
Representative western blots for peEF2 and eEF2. The frontal cortex (FC; left) and the hippocampus (HP; right). Figures represent the peEF_2_/eEF_2_ ratios in the FC and HP. Group conditions are indicated by abbreviations and doses by numbers: Sham (sham treatment), OVX (ovariectomy), Ber (berberine, 5 or 10 mg/kg), Ket (ketanserin, 5 mg/kg) and Imi (imipramine, 30 mg/kg). Symbols represent significant *post hoc* comparisons: Tukey’s HSD, *P < 0.05 vs. OVX only.

The effect of the repeated berberine treatment on the *peEF*_*2*_/*eEF*_*2*_ *ratios* is shown in Fig. 6. There was a significant change across treatment groups in the hippocampus (*P* < 0.05), but not in the frontal cortex (*P* > 0.05). In both regions there were small increases in the peEF_2_/eEF_2_ ratio after ovariectomy, although neither of these effects was statistically significant in *post hoc* comparisons. In the frontal cortex, berberine treatment had a tendency to increase the peEF_2_/eEF_2_ ratio, which was significantly different from the sham control, but not the OVX alone group. Ketanserin did not affect the frontal cortex. In the hippocampus, berberine reversed the slight increase observed in OVX alone mice (Tukey’s HSD, *P* < 0.05 vs. OVX alone). This effect was slightly reversed by ketanserin treatment. Imipramine had no effect on the peEF_2_/eEF_2_ ratio.

### Effect of the repeated berberine treatment on the c-Fos expression

**Figure 7:**
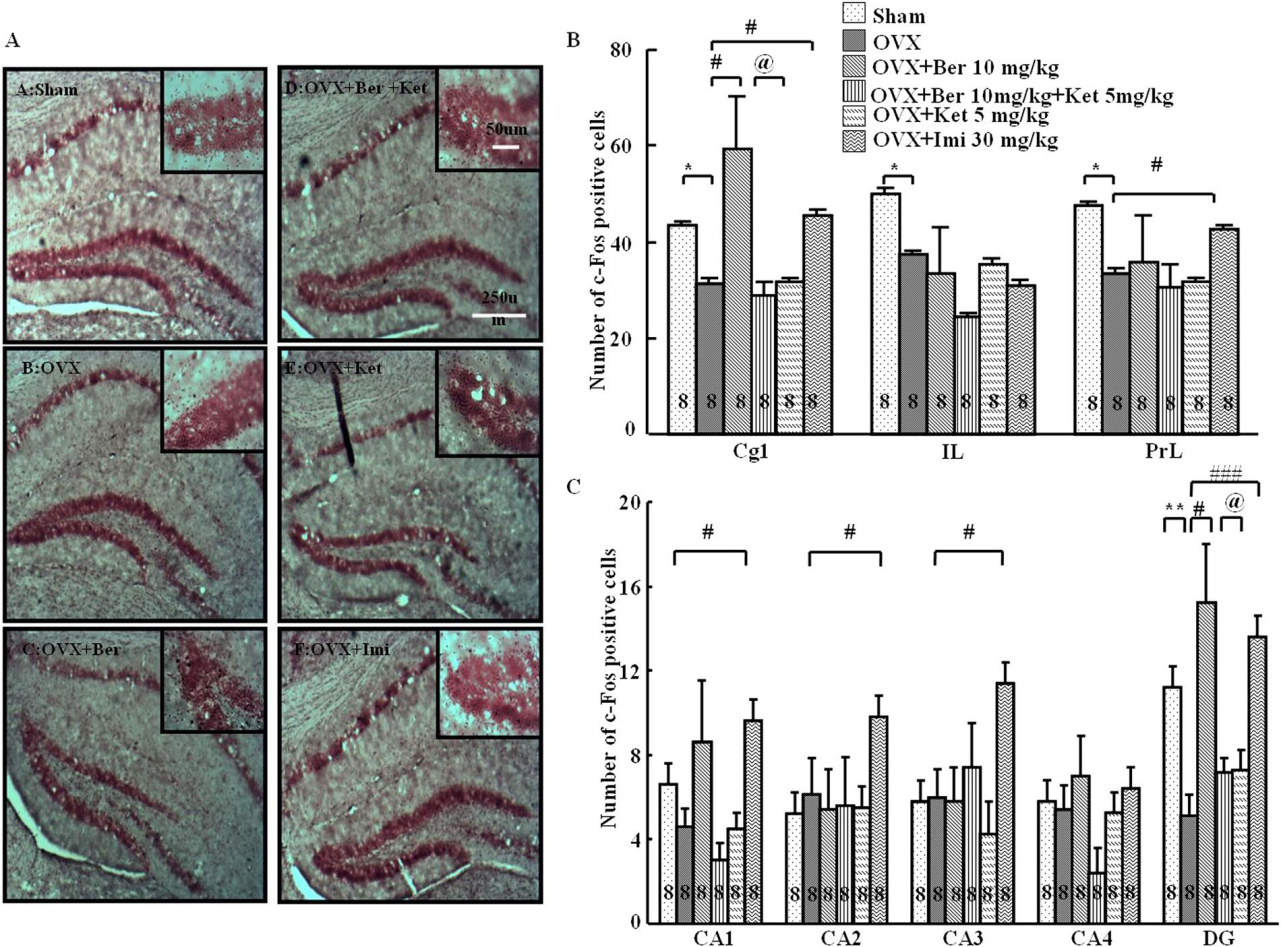
c-Fos-positive cells in frontal cortex and hippocampus. (**A**) Representative sections through the dentate gyrus (inset: greater magnification near the tip of the dentate), showing c-Fos stained cells (dense brown nuclear staining) and counterstaining with neutral red. (**B**) The number of c-Fos positive cells in the subregions of the frontal cortex: cingulate cortex (Cg1), infralimbic cortex (IL), and prelimbic cortex (PrL). (**C**) The number of c-Fos positive cells in the subregions of the hippocampus: cornu ammonis 1–4 (CA1-CA4) and dentate gyrus (DG). Group conditions are indicated by the following letters and abbreviations: (**A**) Sham, sham treatment; (**B**) OVX, ovariectomy only; (**C**) OVX + Ber, ovariectomy and berberine, 10 mg/kg; (**D**) OVX + Ber + Ket, ovariectomy, berberine, 10 mg/kg, and ketanserin, 5 mg/kg; (**E**) OVX + Ket, ovariectomy and ketanserin, 5 mg/kg); and (**F**) OVX + Imi, ovariectomy and imipramine, 30 mg/kg. Symbols represent significant post hoc comparisons: Tukey’s HSD, *P < 0.05, **P < 0.01 vs. Sham; ^#^ *P* < 0.05, ^###^ *P* < 0.001, vs. OVX only; ^@^ *P* < 0.05 vs. OVX + Ber + Ket.

Representative examples of c-Fos stained hippocampal sections are shown in Fig. 7a, and cell counts are depicted in Fig. 7b and c. There were significant differences across treatment groups in both frontocortical and hippocampal subregions. In frontocortical subregions, ovariectomy reduced the c-Fos level in the cingulate cortex (Cg1), the infralimbic cortex (IL) and the prelimbic cortex (PRL). In Cg1, this effect was inhibited by berberine (one-way ANOVA: *P* < 0.01; *P* < 0.05 vs. OVX alone; Tukey’s HSD), an effect that was antagonized by ketanserin (not significantly different from OVX-alone mice but different from OVX/berberine-treated mice, *P* < 0.05). Berberine and ketanserin did not remarkably change the effects of OVX in the IL or PRL. Imipramine reversed the effects of ovariectomy in Cg1 and PrL (*P* < 0.05 vs. OVX alone) but not the IL. Significant differences in c-Fos were found in the dentate gyrus of the hippocampus (*P* < *0*.*001*). Ovariectomy produced a marked reduction in c-Fos only in the dentate gyrus (*P* < 0.05). This effect was reversed by berberine (*P* < 0.05 vs. OVX alone, Tukey’s HSD) and ketanserin (not significantly different from OVX alone but different from OVX/berberine-treated mice, *P* < 0.05). Imipramine increased the c-Fos expression in all areas of the frontal cortex subregions except the CA4 region compared with OVX alone (*P* < 0.05 vs. OVX-alone treated mice, Tukey’s HSD).

## Discussion

Berberine has anti-inflammatory actions^27–31^. However, although it is a constituent of many Chinese traditional medicines, its other potential medical applications remain to be addressed. Our findings indicate that berberine may have antidepressant effects, based on its ability to decrease the immobility time in the forced swimming test in OVX mice without affecting body weight. These results are consistent with previous report indicating that ovariectomy increases the immobility in the forced swimming test, and that Fuzi total alkaloid extract exerted antidepressant-like effects in this model^4^. In addition, there were no body weight changes between OVX animals and sham animals 7 days after ovariectomy, but there were changes 21 days after ovariectomy (data not shown here). Therefore, these results are consistent with other reports that OVX animals gain body weight compared with shams. Shen *et al*. reported that berberine administration for 3 weeks attenuates corticosterone-induced depressive-like behavior in mice^13^. These results also reveal that the effect of berberine may be faster in ovariectomized mice than in corticosterone-treated mice.

The mechanisms that might underlie these effects of berberine were unknown. Therefore, several possible mechanisms were explored in the present experiments. In western blot studies, berberine increased the pCREB/CREB ratio in the frontal cortex, which is reduced by ovariectomy. This is in agreement consistent with previous reports indicating antidepressant treatments increase in CREB phosphorylation^32^. Furthermore, it has been reported that the immunoreactivity for both CREB and pCREB are significantly decreased in the patient frontal cortex with major depressive disorder^33^. These effects are similar to those of an ovariectomy here. On this basis the effects of berberine on CREB pathway were examined in the frontal cortex. These findings suggest that the modulation of CREB signaling may be partially responsible for the behavioral effects of berberine observed here. Interestingly, there was no marked change in the pCREB/CREB ratios in the hippocampus, whereas imipramine altered the pCREB/CREB ratio. The details of these mechanisms that have been initially elucidated here, require further investigation. In addition, hypothalamic-pituitary-adrenal axis (HPA axis) activation and BDNF are related to the antidepressant-like effect of berberine in corticosterone-treated mice^13^. However, the role of the HPA axis in the berberine-induced antidepressant effect in ovariectomized mice also needs further investigation.

Other potential mechanisms may certainly be involved, including those related to fast-acting antidepressants, which have been investigated recently^18^. Accordingly, the effects of berberine on the BDNF-eEF_2_ pathway were also examined. The level of BDNF was reduced in OVX mice in the hippocampus and the frontal cortex. Berberine did not affect reductions in the frontal cortex, but it reversed these changes in the hippocampus. Moreover, these changes in the level of BDNF in the hippocampus were accompanied by reductions in the peEF_2_/eEF_2_ ratio after ovariectomy. Similarly, the herbal medicine Yueju rapidly reduces the peEF2, which leads to the desuppression of BDNF synthesis^18^. Autry *et al*. found that rapid antidepressant-like behavior is regulated by inhibited peEF2 and enhanced BDNF translation^17^. Subchronic treatment with serotonin 5-HT_2C_ receptor antagonists may also produce faster-acting antidepressant effects than many currently available antidepressants^34^. This suggestion was based on effects seen in the chronic forced swimming, chronic mild stress and olfactory bulbectomy models. These actions were linked to the activation of mTOR, BDNF, and eEF_2_ in the frontal cortex. Subchronic treatments also reversed chronic mild stress-induced neuronal atrophy. Similar effects on eEF_2_ were observed in the frontal cortex of OVX mice. Moreover, many of the effects here were shown to be 5-HT-mediated.

5-HT_2_ receptors play an important role in depression. Zen *et al*. reported that ferulic acid exerted an antidepressant-like effect in mice, and this effect was blocked by the 5-HT_2_ receptor antagonist ketanserin. However, the administration of ketanserin alone did not affect the tail suspension test^35^. Therefore, in this study we also examined whether the antidepressant-like effect of berberine was blocked in the forced swimming test. In this study ketanserin partially reversed the effects of berberine on pCREB in the frontal cortex, and BDNF in the hippocampus, although it did not appear to influence eEF_2_. In addition, ketanserin antagonized the effects of berberine on the reductions of c-Fos induced by ovariectomy in the dentate gyrus and cingulate cortex. These results parallel the effects of ovariectomy and these drugs in the forced swimming test, in which ketanserin antagonized the immobility-decreasing effects of berberine on the elevations in immobility time induced by ovariectomy. The effects of ovariectomy have previously been linked to serotonergic alterations, including decreases serotonin 5-HT_2A_ receptors mRNA and protein in rats^36, 37^, and increased 5-HT_2A_ receptor binding after estrogen replacement therapy in postmenopausal women^38, 39^. Kulkarni and Dhir (2007)^40^ reported that berberine may have antidepressant effects in standard models of behavioral despair, including the forced swimming test and the tail suspension test, in otherwise untreated mice. It was suggested that increased levels of monoamines, perhaps, changes in their effects on the nitric oxide levels, were associated with these actions.

In this study, berberine influenced the BDNF-eEF_2_ pathway in the hippocampus, and CREB signaling in the frontal cortex, leading to antidepressant effects in the ovariectomy model of depression. Furthermore, the actions of berberine may be faster than the actions of 2 to 4 weeks of administration of a SSRI or SNRI. In both the hippocampus and the frontal cortex, there were differences found in c-Fos activation, with reductions after ovariectomy, and berberine was able to reverse these reductions. Moreover, these effects were antagonized by ketanserin, which indicated that 5-HT_2_ receptors may partially be involved in these effects. Therefore, berberine should be further explored as a potential new antidepressant treatment.

## Materials and Methods

### Animals

Female ICR mice (from 6 to 10 weeks) were purchased from Jilin University (Changchun, China). Mice were kept in plastic cages (25.5 cm × 15 cm × 14 cm), and maintained in standard laboratory conditions (23 ± 1 °C, a 12 h light dark cycle). The mice were allowed free access to food and water. The surgical procedure for the ovariectomy followed the method outlined by Liu *et al*.^4^, in which the bilateral ovarian resection was performed (9 weeks). Briefly, each mouse (female) was anesthetized with chloral hydrate (10%)^41^. Following an small incision made with a pair of forceps and scissors, the ovary was obtained from the opening in the musculature. A ligature was put around fallopian tube and each ovary before the ovaries, and periovarian fat, were bilateral resection. Sham-operated animals used the same procedure as the OVX mice but without ovarian resection. Behavioral studies were performed during the light phase.

All experiments were carried out in accordance with the Guide for Animal Experimentation of Jilin University. The protocol was approved by the Jilin University Institutional Animal Care and Use Committee.

### Drugs

Berberine (5.0 and 10.0 mg/kg) was purchased from the National Institutes for Food and Drug Control (Beijing, China). Imipramine and ketanserin hydrochloride were purchased from Sigma Aldrich (St. Louis, MO, USA). All drugs were dissolved in saline.

### Experimental Design

For the behavioral portion of the study, Sham ovariectomized animals served as control subjects for the effects of OVX. There were 6 OVX/drug treatment groups, including sham, OVX/saline, OVX/berberine, OVX/berberine/ketanserine, OVX/ketanserine and OVX/imipramine groups. In the behavioral study, two groups of subjects were treated for 7 days with berberine only (5.0 or 10.0 mg/kg i.p.). The OVX/berberine/ketanserine group of mice was treated with berberine (10 mg/kg i.p.) and ketanserin (5.0 mg/kg i.p.). The OVX/ketanserine and OVX/imipramine group served as a positive control, and the animals received imipramine (30 mg/kg, i.p.) as previously reported^4^. Ketanserin was administered 30 min after berberine administration in the berberine/ketanserin group. The dose levels of the berberine and ketanserine used were based on the previous reports^14, 42^.

For the western blotting experiments, all animals were tested behaviorally, and tissues were collected from the prefrontal cortex and hippocampus. In addition, a separate experiment was performed with OVX mice that were treated with imipramine as a positive control. All mice were weighed daily throughout the drug treatments.

### Open field test

Exploratory behavior was performed in an open field test prior to forced swimming test. The test was performed using Hall’s open-field apparatus^38^, and followed the same criterion with slight modification. Mice were put separately in a square acrylic apparatus (48.8 cm × 48.8 cm with 16 cm high walls) with the gray floor, (divided into 16 equal squares). Open field was assessed as the number of grid lines crossed with all of four paws. The frequency of rearing was also measured. The duration of the test was 6 min.

### Forced swimming test

The forced swimmg test was conducted 24 h after the last treatment of drug as previously reported^39^. It was performed in a cylindrical container (25 cm high, 11 cm in diameter) filled with water to 20 cm (25 ± 1 °C). After the test, all mice were dried using a towel and kept warm before going back to home cages. The times of immobility were recorded during swim test (6 min) by an observer blind to treatment conditions, using a captured video of the test. The immobility duration during the last 4 min of the trial was calculated.

### Western blot

Mice were decapitated immediately after forced swimming. The frontocortical and hippocampal tissue was collected, and the protein was extracted using standard procedures. Tissue lysates were immediately assessed for the levels of BDNF, eEF_2_, CREB and pCREB levels using western blot. After separation on 10% SDS-PAGE gels, the proteins were transferred to polyvinylidene difluoride (PVDF) membranes by electroblotting. BDNF, eEF_2_, phosphoeEF_2_, CREB, pCREB and β-actin were immunostained by initial incubation with the following primary antibodies: BDNF (1:1000, rabbit polyclonal; Santa Cruz Bio, CA, USA, #sc546), eEF_2_ (1:1000, rabbit polyclonal; Cell Signaling Technology; #2332), peEF_2_ (1:1000, rabbit polyclonal; Cell Signaling Technology; phospho-eEF_2_ (Thr56)#2331), phosphoCREB-ser133 (1:1000, rabbit polyclonal; Cell Signaling, #CST9197S), CREB (1:1000, rabbit monoclonal; Cell Signaling, #CST9191S) and β-actin (1:2000, mouse monoclonal; Transgen Biotech, #HC201). After the membranes were washes using TBST buffer solution, the respective peroxidase-labeled secondary antibodies (anti-rabbit: 1:400; Protein Tech, #SA00001-2; anti-mouse: 1:5000; ZSBG-Bio, #ZB2305) were used for their incubation. Specific band densities were quantified using the Image J software, and the ratio of intensities of pCREB/CREB, peEF_2_/eEF_2_ and BDNF/β-actin from the same homogenate were calculated^42^.

### C-Fos immunohistochemistry

C-Fos immunohistochemistry was performed as reported by our group^26^. Briefly, all mice were first deeply anesthetized with chloral hydrate (400 mg/kg, i.p.), and brain perfusion with ice cold PBS, followed by 4% paraformaldehyde in PBS was performed. After removal, brains were post-fixed with 30% sucrose. Serial coronal sections (30 µm thick) from different treatment groups were processed in parallel to minimize variation in the immunohistochemical labeling. Free floating sections incubated using 0.6% solution of hydrogen peroxide containing PBS. After rinsing again with PBS buffer, the sections incubated with rabbit polyclonal c-Fos antibody (1:1000, Santa Cruz Biotechnology, #sc-52) solution containing 0.3% Triton X-100, 0.05% sodium azide, and 2% normal goat serum for 72 h at 4 °C. The sections were then whashed and incubated using a secondary antibody (1:400, biotinylated goat anti-rabbit IgG (Vector Laboratories) at dilution in PBS buffer containing 0.3% Triton X-100 for 75 min at room temperature. After rinsing in PBS buffer, the sections were replaced by PBS buffer containing 0.4% avidin-biotinylated horseradish peroxidase complex (Vector Laboratories) for another 75 min. Following washes in PBS buffer and 0.2 M sodium acetate solution, pH 6.0, the reaction was continued using the glucose oxidase-diaminobenzidine-nickel method. The sections were finally washed by 0.2 M sodium acetate solution. Thereafter, the sections were moved to chrome-alum-gelatin-coated slides. We counterstained air-dry the section with neutral red, dehydrated using a graded alcohol series, cleared in xylene and cover slipped. Bilateral c-Fos counting was conducted on a minimum of three representative sections per level in a blind fashion. The positive cells were counted under ×200 magnification from the hippocampus and prefrontal cortex. Separate counting was performed for the prefrontal subregions, the cingulate cortex (Cg1), the infralimbic cortex (IL) and the prelimbic cortex (PrL), according to the atlas of Franklin and Paxinos^43^. Separate counts in the hippocampus were performed in the dentate gyrus, and the CA1, CA2, CA3, and CA4 regions. Using light microscopy, c-Fos positive neurons were identified by dense brown nuclear staining, and captured with a Nikon digital camera (EcLipse 50i Microsope Nikon). The counting c-Fos was blind to experimental conditions. Brain regions were confirmed using the mouse brain atlas^43^. These counts were averaged for five to eight sections in each region for each animal.

### Statistical analyses

All data are expressed as the mean ± S.E.M. We analyzed the data using a two- or one-way analysis of variance (ANOVA). A two-way ANOVA with drug treatment as the between-subjects factor and time as the within-subject factor, was used for the statistical analysis of the body weight data. A one-way ANOVA was used to probe behavioral, immunohistochemistry and western blot data. When significant differences were obtained, *post hoc* comparisons were performed using Tukey’s honestly significant difference test (Tukey’s HSD) to compare treatment groups and sham-treated animals, as well as OVX/berberine- and OVX/berberine/ketanserin-treated animals. *P*-values less than 0.05 were considered significant. Student’s t-test was performed to make comparisons between the imipamine and control group in the western blotting^44–46^.
